# SARS-CoV-2 PCR-positive and PCR-negative cases of pneumonia admitted to the hospital during the peak of COVID-19 pandemic: analysis of in-hospital and post-hospital mortality

**DOI:** 10.1186/s12879-021-06154-z

**Published:** 2021-05-20

**Authors:** Abduzhappar Gaipov, Arnur Gusmanov, Anara Abbay, Yesbolat Sakko, Alpamys Issanov, Kainar Kadyrzhanuly, Zhanar Yermakhanova, Lazzat Aliyeva, Ardak Kashkynbayev, Iklas Moldaliyev, Byron Crape, Antonio Sarria-Santamera

**Affiliations:** 1 Department of Medicine, Nazarbayev University School of Medicine, Kerey and Zhanibek Khans Street 5/1, Room 345, Nur-Sultan city, Kazakhstan; 2 Department of Emergency Medicine, Akhmet Yassawi University Medical Faculty, Turkestan, Kazakhstan; 3 Department of expertise, Social Health Insurance Fund branch of the Turkestan Region, Turkestan, Kazakhstan; 4 Department of Mathematics, Nazarbayev University School of Sciences and Humanities, Nur-Sultan, Kazakhstan; 5 Department of Preventive Medicine, Akhmet Yassawi University Medical Faculty, Turkestan, Kazakhstan

**Keywords:** SARS-CoV-2, COVID-19-like pneumonia, PCR test, In-hospital mortality, Post-hospital mortality, Provisional hospitals, Infectious disease hospitals, Kazakhstan

## Abstract

**Background:**

During the spike of COVID-19 pandemic in Kazakhstan (June-2020), multiple SARS-CoV-2 PCR-test negative pneumonia cases with higher mortality were reported by media. We aimed to study the epidemiologic characteristics of hospitalized PCR-test positive and negative patients with analysis of in-hospital and post-hospital mortality. We also compare the respiratory disease characteristics between 2019 and 2020.

**Methods:**

The study population consist of 17,691 (March–July-2020) and 4600 (March–July-2019) hospitalized patients with respiratory diseases (including COVID-19). The incidence rate, case-fatality rate and survival analysis for overall mortality (in-hospital and post-hospital) were assessed.

**Results:**

The incidence and mortality rates for respiratory diseases were 4-fold and 11-fold higher in 2020 compared to 2019 (877.5 vs 228.2 and 11.2 vs 1.2 per 100,000 respectively). The PCR-positive cases (compared to PCR-negative) had 2-fold higher risk of overall mortality. We observed 24% higher risk of death in males compared to females and in older patients compared to younger ones. Patients residing in rural areas had 66% higher risk of death compared to city residents and being treated in a provisional hospital was associated with 1.9-fold increased mortality compared to those who were treated in infectious disease hospitals.

**Conclusion:**

This is the first study from the Central Asia and Eurasia regions, evaluating the mortality of SARS-CoV-2 PCR-positive and PCR-negative respiratory system diseases during the peak of COVID-19 pandemic. We describe a higher mortality rate for PCR-test positive cases compared to PCR-test negative cases, for males compared to females, for elder patients compared to younger ones and for patients living in rural areas compared to city residents.

**Supplementary Information:**

The online version contains supplementary material available at 10.1186/s12879-021-06154-z.

## Background

More than 61 million people infected, and 1.4 million people died from the COVID-19 pandemic worldwide [1, 2]. These numbers are likely underestimated, and real statistics can be much higher if undiagnosed cases, false-negative cases, and misclassified COVID-19 deaths are counted. The case-fatality rate is probably not associated with country income nor with the healthcare system resources. The top 20 countries with the highest death per million population (> 500 death PMP) are Belgium, San-Marino, Peru, Andorra, Spain, Italy, Argentina, UK, Brazil, USA, Chile, Mexico, Bolivia, France, Ecuador, North Macedonia, Bosnia and Herzegovina, Montenegro, Colombia, Czechia [1]. To date, the entire world is attempting to prevent and decrease the incidence to manageable numbers; however, high contagiousness and rapid spread of COVID-19 has led to an increasing number of infected patients, which have not been seen in previous SARS viral infections [3].

Kazakhstan is a middle-income country with 18 million population, with 168,083 confirmed cases of COVID-19 and 2417 deaths (93 death PMP) as of November 26, 2020 [4]. There was a sharp rise of atypical pneumonia of unknown etiology in June, 2020 with clinical symptoms, computed tomography findings and epidemiological characteristics similar to COVID-19, but negative PCR results [5, 6], which was later defined as “COVID-19-like pneumonia”. The “COVID-19-like pneumonia” data registration began on August 1, 2020, and to date, officially 41,159 cases of COVID-19-like pneumonia with 427 deaths have been reported [5]. However, the official statistics could be underestimated given the “COVID-19 like pneumonia” cases before August 2020 were not documented. The reasons for PCR-negative test results for “COVID-19-like pneumonia” is likely laboratory false-negative cases, although recent studies reported the possibility of undetectable PCR test in some cases [7–11].

True positivity of the SARS-CoV-2 PCR test is extremely important to make the correct diagnosis, timely suspect infected cases, to calculate the reproduction number and forecast the spread of infection [12, 13]. International and local guidelines of diagnosis and treatment of COVID-19 and healthcare organizational measures such as preventive and quarantine actions, triage and transfer of patients with suspected and confirmed cases, heavily rely on the results of PCR tests [14, 15]. The Kazakhstan Republican Center of Healthcare Development created a national protocol for diagnosis and management of COVID-19, based on clinical characteristics, PCR-test results and chest-CT results [16, 17]. Furthermore, PCR-test results are essential for initiation of early treatment to prevent the development of severe illness and to reduce mortality. Otherwise, in the early stages of the disease, most false-negative cases are managed symptomatically, resulting in an increase of fatal outcomes, especially during the peak of the outbreak.

The majority of countries faced with the peak of the COVID-19 pandemic experienced disastrous issues in healthcare, including shortages of hospital beds, medical staff and diagnostic tools (PCR-tests and chest CT or X-ray), deficient supplies of etiological medications, personal protective equipment (PPE), oxygen supply and lung ventilation devices, and even confronted with a collapse of healthcare system [18, 19]. In Kazakhstan, the most impactful outbreak of COVID-19 pandemic was recorded in the Turkistan oblast (southern region of Kazakhstan) between June, 2020 - August, 2020, notable for a surge of atypical pneumonia cases with negative PCR-testing for COVID-19 [6]. The medical community and mass media repeatedly expressed concerns about the large numbers of PCR-negative pneumonia cases, shortage of medical care and high mortality among hospitalized patients with pneumonia [5]. However, there was no well-defined data describing the incidence and mortality rate among patients with “COVID-19 like pneumonia”. Furthermore, there was no clear statistics on post-hospital mortality even for PCR positive and negative cases of pneumonia.

Given the previously mentioned data gap, we aimed to study the epidemiologic disparities of hospitalized patients with SARS-CoV-2 PCR-positive and PCR-negative test results, with analysis of in-hospital and post-hospital mortality. We also aimed to compare incidence and mortality rates from respiratory diseases among hospitalized patients between the period of March–July 2019 and 2020.

## Methods

### Study population and data sources

The study population consisted of all hospitalized patients with respiratory diseases (including COVID-19) according to the International Statistical Classification of Diseases and Related Health Problems (ICD-10) from March to July 2019 and March to July 2020 in Turkestan oblast, Kazakhstan. The following ICD-10 codes were included to the study: **J00-J06** (acute upper respiratory infections), **J09-J18** (influenza and pneumonia), **J20-J22** (other acute lower respiratory infections), **J40-J47** (chronic lower respiratory diseases), **J96-J99** (other diseases of the respiratory system), **B34** (viral infection of unspecified site), **Z20** (contact with and “suspected” exposure to communicable diseases), **U07.1** (virus-specified COVID-19) and **U07.2** (virus-unspecified COVID-19).

The raw data was retrieved from the Unified National Electronic Health System (UNEHS) linked with the records to the “Electronic Registry of Inpatients” which included data on dates of admission and discharge, ICD-10 codes, PCR-test dates and results, discharge outcomes and some demographic data. The overall mortality (in-hospital and post-hospital death) statistics were obtained independently from the “Registry of Attached Population” and linked to the hospitalized patients via the Population Registry Number (RPN-ID); each date of death followed after date of discharge from the hospital considered as post-hospital mortality. The population census of the Turkestan oblast including all cities and rural areas (2,016,100 persons) was obtained from the State Statistics Committee .

### SARS-CoV-2 infection detection method

The SARS-CoV-2 infection confirmation was done using real-time quantitative PCR on nasopharyngeal swabs with BGI-kit (Beijing Genomics Institute, Shenzhen, China) in special defined regional laboratory settings.

### Exposures and covariates

Demographic characteristics included age, sex and residency setting (rural and urban areas). Age was categorized as “<20 years old (y.o.)”, “20–29 y.o.”, “30–39 y.o.”, “40–49 y.o.”, “50–59 y.o.”, “60–69 y.o.”, “> 69 y.o.”. Variables related to hospitalization, namely, month of hospitalization, duration of stay in days, outcome at discharge (“without change”, “recovery”, “improvement”, “deterioration”, “in-hospital death” and “post-hospital death”) and type of medical organization were collected for both 2019 and 2020 cohorts. Healthcare organizations, depending on location and classification, were categorized as a city, oblast, and regional (rayon) hospitals, and other medical or non-medical (temporary) organizations. All hospitals involved in admission of COVID-19 patients during the 2020 pandemic period were classified as quarantine, provisional and infectious disease hospitals as defined by the Ministry of Healthcare of the Republic of Kazakhstan. Depending on the availability of PCR-testing and their results (in the 2020 cohort), all admitted patients were categorized as SARS-CoV-2 PCR-test positive cases (if at least one test was positive), PCR-test negative cases and PCR-test unknown cases (if the PCR-testing were not performed or results not available).

### Definition of quarantine, provisional and infectious disease hospitals

The regional (rayon), city, and oblast hospitals can be characterized as a primary, secondary, and tertiary care hospitals based on their medical services. Most of the hospitals as well as some non-medical organizations, such as hotels, university and college dormitories were assigned to be a quarantine hospital from March to June 2020. Admission criteria to quarantine hospitals were asymptomatic subjects being contacted with confirmed and/or suspected cases of COVID-19 infected patients or being entered to the country from the epidemic countries/zones. Beginning from June, when the number of pneumonia cases increased, all hospital were transferred to whether provisional or infectious diseases hospitals. Admission criteria to the provisional hospitals were symptomatic patients with flu-like symptoms, pneumonia and unknown SARS-CoV-2 PCR-test, while admission criteria to infectious disease hospitals – symptomatic patients with pneumonia with positive SARS-CoV-2 PCR test.

### Outcome assessment

The incidence, mortality and case-fatality rates were assessed. Incidence and mortality rates were calculated for each year using the number of newly-diagnosed patients and deaths, and population size. The case-fatality rate was calculated by the number of deaths divided by the number of newly-diagnosed cases. The incidence was compared by year of admission. All-cause mortality was divided into in-hospital and post-hospital mortality, which was used for identification of associated risk factors among admissions in 2020.

The start of the follow-up was the date of hospital admission, and patients were followed until death or end of follow-up period (August 30th, 2020). Two outcome variables were of interest for survival analysis - in-hospital mortality (time from hospital admission to hospital discharge) and overall (combined in-hospital and post-hospital) mortality (time from hospital admission to death any time until August 30th, 2020). Censoring for in-hospital mortality survival analysis was taken on the date of discharge from the hospital, and for combined mortality it was on August 30th, 2020.

### Statistical analysis

For each group of diagnoses absolute numbers of hospitalizations and deaths, incidence and mortality rates per 100,000, case-fatality rates were reported by year. Absolute and relative frequencies were reported for categorical variables. Means and standard deviations were used to describe continuous variables, whereas skewed continuous variables were characterized by medians and interquartile ranges (IQR). Parametric bivariate analysis (Pearson’s Chi-squared, two-sample t-test, ANOVA) was utilized to assess associations of demographic and disease-related characteristics with outcome variables. The Kaplan-Meier survival curves were plotted for PCR-test results. Cox’s Proportional Hazards Models were fit with epidemiologically and statistically significant co-variables using backward stepwise selection. The assumption of proportional hazards for different groups was tested using log-log plots.

We conducted sensitivity analysis to evaluate the robustness of our main findings. The association between overall mortality (in-hospital and post-hospital) and socio-demographic parameters were examined in a subgroup of patients admitted to only provisional and infectious disease hospitals (excluding patients who were quarantined).

The significance level of 5% (α < 0.05) was taken. All statistical analyses were performed using STATA 16.0 statistical software [20]. The study was approved by the Institutional Review Ethics Committee (NU-IREC 203/29112019) with exemption from informed consent.

## Results

### Comparison of demographic and disease-related characteristics for hospital admissions by the PCR-test results

**Fig. 1:**
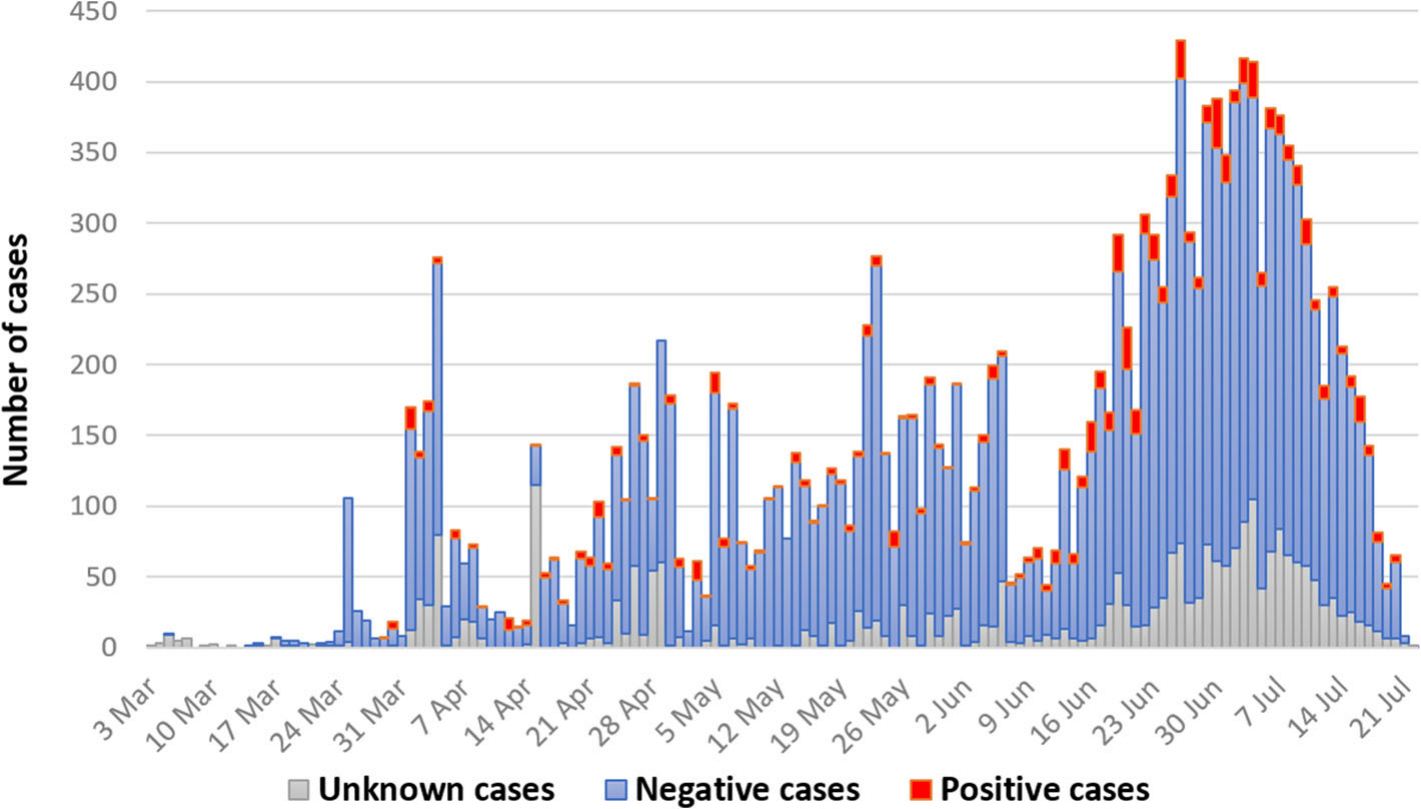
Incidence of SARS-CoV-2 PCR-test positive, negative and unknown cases

In Table 1 the bivariate analysis between the demographic characteristics of patients admitted in 2020 and the PCR test results on COVID-19 is shown. In the cohort, 4.6% of patients had tested positive at least once, while 81.5% had tested negative and 14.3% not tested. Among the 6993 patients with “Influenza and Pneumonia” (ICD-10 J09-J18), 84.8% (*n* = 5930) have SARS-CoV-2 PCR-test negative result and considered as “COVID-19-like pneumonia”. Patients with a positive test had a higher median number of days spent in hospital (median 13, IQR 4–16) compared to the negatively-tested (median 3, IQR 2–6) and not tested patients (median 3, IQR 2–6, *p* < 0.001). The number of patients with positive results was higher in June and July (6.0 and 4.3%) compared to the period between March and May (less than 3.9%, p < 0.001). Besides all patients with virus-specified COVID-19 diagnosis (U07.1) with positive test results, 54.5% of patients with the diagnosis of viral infection of unspecified site (B34) were tested positive, which was significantly higher compared to other health problems. The highest proportion of positively-tested patients was in infectious disease hospitals (56.2%) compared to quarantine (0.2%) and provisional (1.7%) hospitals (*p* < 0.001), also, in city hospitals (14.1%) compared to rural areas such rayon (6.0%) and oblast (1.1%) hospitals, and other medical (1.9%) and non-medical (0.1%) organizations (p < 0.001). The highest proportion of patients with unknown test results were in other medical organizations - 58.1%. The Incidence of cases with positive, negative and unknown test results is shown in Fig. 1.

**Table 1:** Socio-demographic and disease-related variables for different results of SARS-CoV-2 PCR test

Variables	PCR-test negative cases ***n*** = 14,356 (81.2%)	PCR-test positive cases ***n*** = 805 (4.**5**%)	PCR-test unknown cases ***n*** = 2530 (14.3%)	***p***-value
**Age, Mean (**± **SD)**	42.8 (± 18.8)	45.1 (± 17.6)	44.3 (±17.8)	< 0.001^1^
Age, N (%)				< 0.001^2^
< 20	1426 (87.6)	50 (3.1)	151 (9.3)	
20–29	2215 (80.4)	121 (4.4)	418 (15.2)	
30–39	2607 (80.8)	144 (4.5)	475 (14.7)	
40–49	2438 (80.7)	118 (3.9)	464 (15.4)	
50–59	2720 (80.5)	190 (5.6)	469 (13.9)	
60–69	1934 (80.0)	122 (5.0)	363 (15.0)	
> 69	1016 (80.2)	60 (4.7)	190 (15.0)	
**Sex, N (%)**				0.002^2^
Female	7082 (82.2)	379 (4.4)	1156 (13.4)	
Male	7274 (80.2)	426 (4.7)	1374 (15.1)	
**Residency, N (%)**				< 0.001^2^
Rural	7978 (82.6)	429 (4.4)	1252 (13.0)	
City	6378 (79.4)	376 (4.7)	1278 (15.9)	
**Month of admission, N (%)**				< 0.001^2^
March	210 (79.5)	6 (2.3)	48 (18.2)	
April	2140 (75.9)	109 (3.9)	571 (20.3)	
May	3271 (89.8)	123 (3.4)	250 (6.8)	
June	4672 (81.1)	344 (6.0)	741 (12.9)	
July	4063 (78.0)	223 (4.3)	920 (17.7)	
**Number of days in hospital, median (IQR)**	3 (2–6)	13 (4–16)	3 (2–6)	< 0.001^1^
**Final diagnoses, N (%)**				< 0.001^2^
**J00-J06:** Acute upper respiratory infections	1028 (95.6)	2 (0.2)	45 (4.2)	
**J09-J18:** Influenza and pneumonia	5930 (84.8)	61 (0.9)	1002 (14.3)	
**J20-J22:** Other acute lower respiratory infections	70 (85.4)	0 (0.0)	12 (14.6)	
**J40-J47:** Chronic lower respiratory diseases	12 (100.0)	0 (0.0)	0 (0.0)	
**J96-J99:** Other diseases of the respiratory system	91 (68.9)	4 (3.0)	37 (28.0)	
**B34:** Viral infection of unspecified site	282 (26.2)	586 (54.5)	208 (19.3)	
**Z20:** Contact with and (suspected) exposure to communicable diseases	5517 (86.7)	7 (0.1)	842 (13.2)	
**U07.1:** COVID-19 (virus specified)	0 (0.0)	145 (100.0)	0 (0.0)	
**U07.2:** COVID-19 (virus unspecified)	1425 (78.8)	0 (0.0)	384 (21.2)	
**Clinical outcome at discharge**				< 0.001^2^
Without change	1092 (68.6)	121 (7.6)	379 (23.8)	
Recovery	3646 (76.1)	427 (8.9)	717 (15.0)	
In-hospital death	139 (61.5)	32 (14.2)	55 (24.3)	
Improvement	9436 (85.6)	216 (2.0)	1376 (12.5)	
Deterioration	43 (78.2)	9 (16.4)	3 (5.4)	
**Post-hospital mortality**	245 (1.7)	9 (1.1)	40 (1.6)	0.42
**Type of Hospital**				< 0.001^2^
Quarantine	5490 (86.7)	13 (0.2)	828 (13.1)	
Provisional	8584 (83.7)	172 (1.7)	1501 (14.6)	
Infectious diseases	282 (25.6)	620 (56.2)	201 (18.2)	
**Type of Healthcare organization**				< 0.001^2^
City hospitals	1296 (69.6)	262 (14.1)	303 (16.3)	
Oblast hospitals	226 (81.6)	3 (1.1)	48 (17.3)	
Rayon hospitals	7262 (88.7)	491 (6.0)	436 (5.3)	
Other med organizations	918 (40.0)	44 (1.9)	1332 (58.1)	
Non-medical organizations (temporary)	4654 (91.8)	5 (0.1)	411 (8.1)	
**Number of PCR-tests**				< 0.001^2^
0 (not in hospital)	47 (1.7)	102 (3.8)	2530 (94.4)	
1	13,134 (98.1)	259 (1.9)	0 (0)	
2	1002 (79.0)	266 (21.0)	0 (0)	
3	157 (55.5)	126 (44.5)	0 (0)	
> 4	16 (23.5)	52 (76.5)	0 (0)	

^1^ ANOVA, ^2^ Pearson’s Chi-squared test

### Comparison of demographic and disease-related characteristics for hospital admissions between in-hospital and post-hospital death groups

Bivariate analysis between demographic variables of admissions in 2020 and mortality - in-hospital and post-hospital is presented in Table 2. Patients who died following discharge were older on average (64.0 ± 13.0) in comparison with alive patients (42.5 ± 18.5) and those who died in hospital (61.4 ± 11.4, *p* < 0.001). Both in-hospital and post-hospital mortality were more frequent among those older than 50 years with the highest proportions of deaths in the > 69 y.o. age subgroup (4.3 and 8.2%, respectively). Higher proportion of patients died after discharge in rural areas compared to city residents (2.2% vs 1.0%, *p* < 0.001). Patients in the post-hospital death group had fewer days spent in the hospital (median 2, IQR 2–4) compared to patients in alive (median 3, IQR 2–7) and in-hospital death groups (median 3, IQR 2–7, p < 0.001). In-hospital deaths were highest among patients with chronic lower respiratory diseases (16.7%) and other diseases of the respiratory system (10.6%) compared to those with other disorders. Post-hospital deaths were registered more frequently in admissions for U07.2 (virus-unspecified COVID-19) ICD-10 code (4.8%) and J09-J18 (influenza and pneumonia) ICD-10 code (2.6%) in comparison with other diagnoses, and probably they were PCR-test negative for SARS-CoV2. In-hospital deaths were highest among patients admitted to infectious disease hospital (3.3%), whereas post-hospital deaths were highest in patients discharged from the provisional hospitals (2.8%, *p* < 0.001) compared to other respective categories. In-hospital and post-hospital deaths were higher in city (2.7 and 2.4%, in turn) and rayon hospitals (1.7 and 2.7%, respectively) compared to oblast hospitals, and other medical and non-medical organizations.

**Table 2:** Socio-demographic and disease-related variables for different types of mortality (in-hospital and post-hospital)

Variables	Alive (***n*** = 17,171) (97.1%)	In-hospital death (***n*** = 226) (1.3%)	Post-hospital death (***n*** = 294) (1.7%)	p-value
**Age, mean (sd)**	42.5 (18.5)	61.4 (11.4)	64.0 (13.0)	< 0.001^1^
Age, N (%)				< 0.001^2^
< 20	1626 (99.9)	0 (0.0)	1 (0.1)	
20–29	2746 (99.7)	2 (0.1)	6 (0.2)	
30–39	3213 (99.6)	7 (0.2)	6 (0.2)	
40–49	2975 (98.5)	23 (0.8)	22 (0.7)	
50–59	3269 (96.7)	54 (1.6)	56 (1.7)	
60–69	2233 (92.3)	85 (3.5)	101 (4.2)	
> 69	1109 (87.5)	55 (4.3)	102 (8.1)	
**Sex, N (%)**				0.94^2^
Female	8364 (97.1)	108 (1.2)	145 (1.7)	
Male	8807 (97.1)	118 (1.3)	149 (1.6)	
**Residency, N (%)**				< 0.001^2^
Rural	9331 (96.6)	116 (1.2)	212 (2.2)	
City	7840 (97.6)	110 (1.4)	82 (1.0)	
**Month of admission, N (%)**				< 0.001^2^
March	263 (99.6)	1 (0.4)	0 (0)	
April	2811 (99.7)	0 (0)	9 (0.3)	
May	3633 (99.7)	1 (0.1)	10 (0.3)	
June	5453 (94.7)	132 (2.3)	172 (3.0)	
July	5011 (96.2)	92 (1.8)	103 (2.0)	
**Number of days in hospital, median (IQR)**	3 (2–7)	3 (2–7)	2 (2–4)	< 0.001^1^
**Final diagnoses, N (%)**				< 0.001^2^
**J00-J06:** Acute upper respiratory infections	1065 (99.1)	0 (0)	10 (0.9)	
**J09-J18**: Influenza and pneumonia	6673 (95.4)	141 (2.0)	179 (2.6)	
**J20-J22:** Other acute lower respiratory infections	82 (100.0)	0 (0)	0 (0)	
**J40-J47:** Chronic lower respiratory diseases	9 (75.0)	2 (16.7)	1 (8.3)	
**J96-J99:** Other diseases of the respiratory system	117 (88.6)	14 (10.6)	1 (0.8)	
**B34:** Viral infection of unspecified site	1034 (96.1)	32 (3.0)	10 (0.9)	
**Z20:** Contact with and (suspected) exposure to communicable diseases	6361 (99.9)	0 (0.0)	5 (0.1)	
**U07.1:** COVID-19 (virus specified)	139 (95.9)	6 (4.1)	0 (0.0)	
**U07.2:** COVID-19 (virus unspecified)	1691 (93.5)	31 (1.7)	87 (4.8)	
**Type of Hospital**				< 0.001^2^
Quarantine	6327 (99.9)	0 (0)	4 (0.1)	
Provisional	9786 (95.4)	189 (1.8)	282 (2.8)	
Infectious diseases	1058 (95.9)	37 (3.3)	8 (0.7)	
**Type of Healthcare organization**				< 0.001^2^
City hospitals	1765 (94.8)	51 (2.7)	45 (2.4)	
Oblast hospitals	275 (99.3)	2 (0.7)	0 (0)	
Rayon hospitals	7830 (95.6)	139 (1.7)	220 (2.7)	
Other med organizations	2233 (97.3)	34 (1.5)	28 (1.2)	
Non-medical organizations	5068 (99.98)	0 (0.0)	1 (0.02)	
**PCR-test result**				< 0.001
negative	13,972 (97.3)	139 (1.0)	245 (1.7)	
positive	764 (94.9)	32 (4.0)	9 (1.1)	
unknown	2435 (96.2)	55 (2.2)	40 (1.6)	
**Number of PCR-tests**				0.603 2
0	2555 (95.4)	79 (2.9)	45 (1.7)	
1	13,031 (97.3)	132 (1.0)	230 (1.7)	
2	1240 (97.8)	14 (1.1)	14 (1.1)	
3	277 (97.9)	1 (0.3)	5 (1.8)	
> 4	68 (100.0)	0 (0)	0 (0)	

^1^ ANOVA, ^2^ Pearson’s Chi-squared test

### Organizational actions of healthcare system during the COVID-19 pandemic

**Fig. 2:**
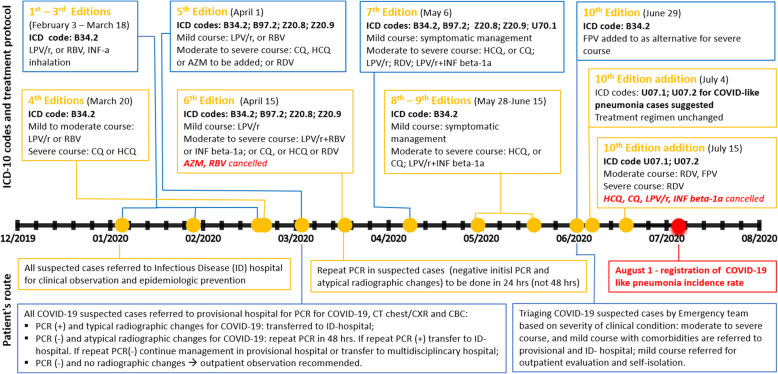
Timeline showing changes in diagnosis, ICD-10 coding and management of COVID-19 guidelines in Republic of Kazakhstan

The national healthcare system underwent reorganizational changes in response to pandemic, which included development of COVID-19 protocol, rearrangement of all available medical and non-medical facilities to quarantine, provisional and infectious diseases hospitals, obtaining supply of PPEs and medications, and many other healthcare activities. The national protocol on COVID-19 with timeline changes is demonstrated in Fig. 2, which was revised more than ten times from February to July 2020 with updated recommendations for diagnosis, ICD-coding, management protocols and triaging guidelines based on PCR-test results and clinical condition severity. Per national guideline recommendation, February to June 29th, PCR-test positive cases were routed to infectious diseases hospitals, whereas PCR-test negative cases stayed in provisional hospitals. Beginning from July 4th, “COVID-like pneumonia” cases started to be coded and triaging of COVID-19 suspected cases was based on clinical course severity - moderate to severe cases transferred/admitted to infectious disease and provisional hospitals independently of PCR-tests results.

**Fig. 3:**
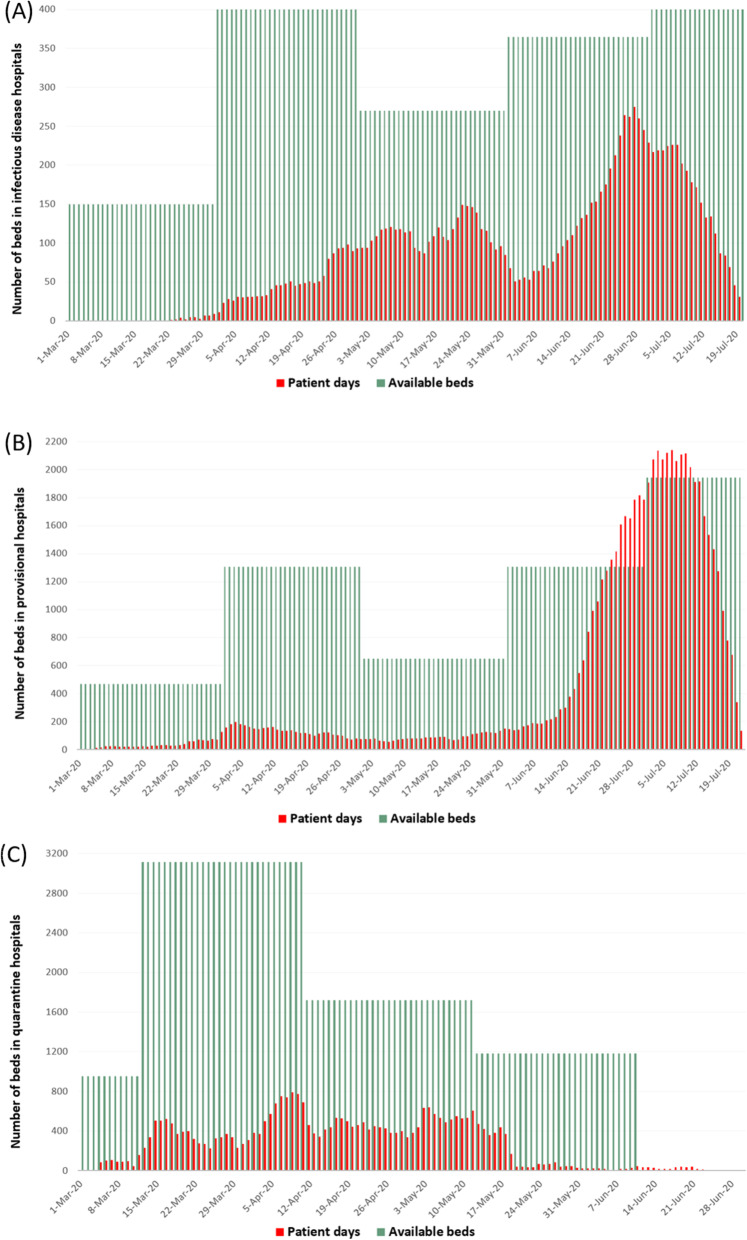
Dynamic change on hospital beds and admitted patients in infectious disease hospitals (**a**), provisional hospital (**b**) and quarantine hospitals (**c**)

Dynamic data on hospital beds and number of admitted patients to infectious disease, provisional and quarantine hospitals depicted in Fig. 3. During the COVID-19 pandemic, there were up to 18 quarantine hospitals with a maximum of 3116 beds, up to 32 provisional hospitals with 1941 beds and up to 10 ID-hospitals with 440 beds. The overall number of beds in quarantine and ID-hospitals was sufficient during the entire period of COVID-19 pandemic, however, the number of patient-days exceeded available beds in provisional hospitals during the highest peak of pandemic between June 20th to July 20th, 2020.

### Mortality assessment during the COVID-19 pandemic

**Fig. 4:**
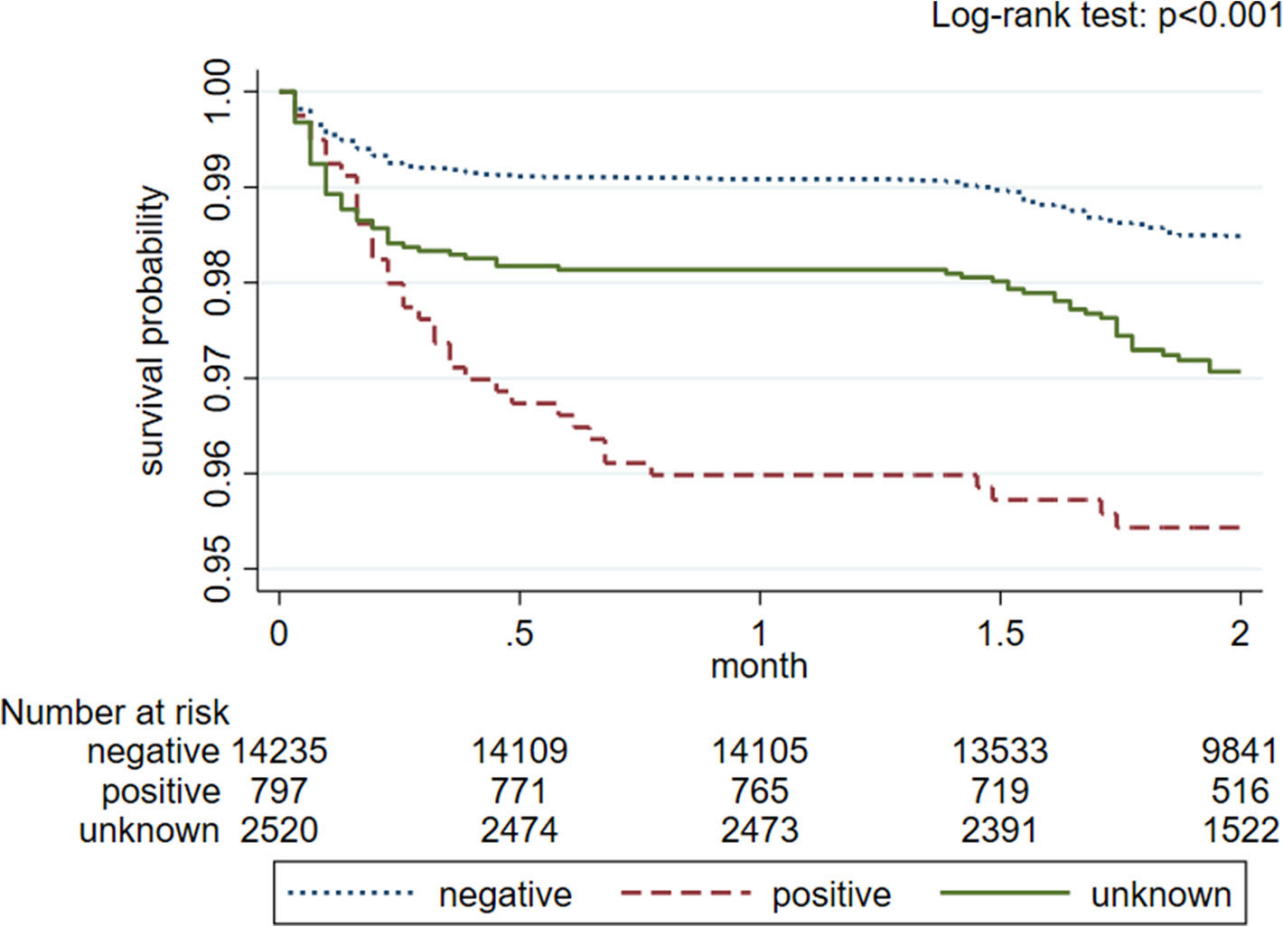
Unadjusted overall (combined in-hospital and post-hospital) survival probability by SARS-CoV-2 PCR-test results

There were 520 deaths reported - 226 cases in-hospital and 294 cases post-hospital deaths, with median 92 [IQR 61–148] days of overall follow-up. The post-hospital deaths had occurred with a decremental trend during the first 2 weeks of post-discharge period (Supplement Figure 1). Kaplan-Meier analysis (Fig. 4) showed that overall (combined in-hospital and post-hospital) patients with PCR-test negative results had a higher probability of survival compared to PCR-test positive and unknown cases. Similar trend was observed for in-hospital mortality (Supplement Figure 2). The cumulative incidence of post-hospital mortality was higher for PCR-test negative cases compared to PCR-test positive cases (Supplement Figure 3).

In Cox proportional hazard ratio regression analysis (Table 3), the PCR-positive cases (compared to PCR-negative cases) had 2-fold higher risk of crude overall mortality and remained similar after adjustments. Compared to patients aged younger than 40 y.o. (reference), patients aged 40–59 y.o. and over 60 y.o. had 10-fold and 53-fold higher risk of overall death, respectively in both univariate and multivariate Cox regression analyses (Table 3). Males had 24% higher risk of overall death compared to females in multivariable Cox regression models. Patients residing in rural areas had 66% higher risk of overall death compared to city residents and remained significant after adjustments. Being treated in a provisional hospital was associated with 1.9-fold increase in overall mortality compared to those who were treated in infectious disease hospitals.

**Table 3:** Association between overall mortality (in-hospital and post-hospital) and socio-demographic parameters using unadjusted (crude) and adjusted Cox proportional hazard regression analysis

Overall (combined in-hospital and post-hospital) mortality				
Variable	Bivariate analysis		Multivariable model	
**Variable**	**crude HR (95% CI)**	***P*** **value**	**adjusted HR (95% CI)**^a^	**P value**
**Sex**		0.13		0.017
Female	Ref.		Ref.	
Male	0.87 (0.73–1.04)		1.24 (1.04–1.48)	
**Age category**				
< 40	Ref.	Ref.	Ref.	Ref.
40–59	10.64 (6.79–16.66)	< 0.001	10.61 (6.77–16.63)	< 0.001
> =60	53.55 (34.66–82.73)	< 0.001	53.97 (34.85–83.56)	< 0.001
**Residency**		< 0.001		0.002
City	Ref.		Ref.	
Rural	1.66 (1.38–1.99)		1.33 (1.11–1.60)	
**PCR-test results**				
negative	Ref.	Ref.	Ref.	Ref.
positive	2.05 (1.49–2.84)	< 0.001	1.83 (1.32–2.53)	< 0.001
unknown	1.38 (1.09–1.75)	0.006	1.40 (1.11–1.78)	0.005
**Hospital profile**		< 0.001	–	–
Infectious disease	Ref.			
Provisional	1.95 (1.41–2.70)			

^a^ Adjusted for: Sex, age, residency, PCR-test results

Similar trends were observed for associations between in-hospital mortality (Supplement Table 1) and post-hospital mortality (Supplement Table 2) with socio-demographic parameters. However, association was lost between post-hospital mortality and SARS-CoV-2 PCR-test results after competing risk regression models (Supplement Table 2). In subgroup analysis, when we excluded from the main cohort those patients (n = 6331) who were admitted to the quarantine hospitals the results remained relatively similar (Supplement Table 3).

### Comparison of morbidity and mortality of hospitalized patients with respiratory diseases between march–august 2019 and 2020

In the Turkestan oblast 4600 and 17,691 hospital admissions with respiratory system diseases (ICD-10 codes: J00-J99, B34, Z20, U07) were registered during the same months from March to July of 2019 and 2020, respectively. The main measures of morbidity and mortality are presented in Table 4. The incidence rate of above-mentioned conditions was 4-fold higher in 2020 compared to 2019 (877.5 vs 228.2 cases per 100,000). The increase in incidence rate was mainly due to the rise of influenza and pneumonia (346.9 cases per 100,000), exposure to communicable diseases (315.7 cases per 100,000) and COVID-19 (virus-specified - 7.2 cases per 100,000 and virus-unspecified - 89.7 cases per 100,000). For the cohort of 2020, rates of in-hospital mortality accounted for 11.2 per 100,000, while in 2019, in-hospital mortality rate was 1.2 deaths per 100,000. The increase in mortality rate in the 2020 cohort (7.0 deaths per 100,000) was due to influenza and pneumonia.

**Table 4:** Incidence rate and mortality rate of hospitalized patients with respiratory diseases in March–July 2020 and 2019

ICD-10 code of admission’s final diagnosis	March – July 2020					March – July 2019				
	Admitted cases	Incidence rate (per 100,000)	In-hospital deaths	Mortality rate (per 100,000)	Case-fatality rate	Admitted cases	Incidence rate (per 100,000)	In-hospital deaths	Mortality rate (per 100,000)	Case-fatality rate
**J00-J06:** Acute upper respiratory infections	1075	53.3	0	0	0	2532	125.6	1	0.05	0.0004
**J09-J18:** Influenza and pneumonia	6993	346.9	141	7.0	0.02	737	36.6	7	0.35	0.01
**J20-J22:** Other acute lower respiratory infections	82	4.1	0	0	0	38	1.9	0	0	0
**J40-J47:** Chronic lower respiratory diseases	12	0.6	2	0.1	0.17	457	22.7	11	0.55	0.02
**J96-J99:** Other diseases of the respiratory system	132	6.5	14	0.7	0.11	805	39.9	5	0.2	0.01
**B34:** Viral infection of unspecified site	1076	53.4	32	1.6	0.03	21	1.0	0	0	0
**Z20:** Contact with and (suspected) exposure to communicable diseases	6366	315.7	0	0	0	10	0.5	0	0	0
**U07.1:** COVID-19 (virus specified)	145	7.2	6	0.3	0.04	–	–	–	–	–
**U07.2:** COVID-19 (virus unspecified)	1809	89.7	31	1.5	0.02	–	–	–	–	–
**Total**	**17,691**	**877.5**	**226**	**11.2**	**0.01**	**4600**	**228.2**	**24**	**1.2**	**0.005**

Comparison of demographic and disease-related characteristics for hospitalizations between March–August 2019 and 2020 is shown in Supplement Table 4. Patients admitted in 2020 were older (43.2 ± 18.6 vs 28.5 ± 23.1, p < 0.001) and stayed fewer days in hospitals (median 3, IQR 2–6 vs median 7, IQR 5–9, p < 0.001) compared to those in 2019. The majority of admissions in 2019 were in rural areas (69.3%), while in 2020 the urban-rural ratio leveled off.

In-hospital survival analysis of 2019 cohort, patients over 60 y.o. had higher mortality compared to younger ones, however there were no associations between sex and residency with risk of in-hospital mortality (Supplement Table 5).

## Discussion

To the best of our knowledge, this is the first study among Central Asian and Eurasian countries, reporting in-hospital and post-hospital mortality of patients admitted with respiratory diseases during the peak of COVID-19 pandemic in March–July 2020, including diagnosis of SARS-CoV-2 PCR-positive and PCR-negative pneumonia as well as other respiratory system diseases. We found that the incidence rate and mortality rate per hundred thousand patients for respiratory diseases were 4-fold and 11-fold higher in March–July 2020 compared to March–July 2019, respectively. During that period 84.8% of those cases were diagnosed as “COVID-19-like pneumonia”, a term suggested for cases with similar epidemiological and clinical features of COVID-19 but that were PCR-test negative for SARS-Cov-2. Survival and cox regression analysis showed that SARS-CoV2 PCR-test positive cases had 2-fold higher risk of death compared to PCR-test negative cases. These results should be interpreted cautiously, because PCR-negative case population may include a higher proportion of other respiratory diseases than COVID-19 pneumonia.

Survival was also statistically significant lower in males compared to females, in elderly patients compared to younger ones, and in patients living in rural areas compared to city residents. Patients treated at provisional hospitals had less chance of survival compared to those treated at infectious disease hospitals.

Our current findings suggest that males have higher risk of death compared to females, and elderly patients compared to younger ones is consistent with worldwide data [21–23]. It is also well known that elderly patients have more comorbidities and pose a higher risk for COVID-19 related complications and mortality [23, 24].

The higher risk of serious illness and mortality among rural population during the COVID-19 pandemic were reported by investigators [25–27]. Similarly, we described the greater chance of survival among city residents compared to residents of rural regions. Our data showed that 69.7% of rural residents were treated at the rayon hospitals, while 78.8% of city residents were treated at the city hospitals. We suspect the advantages of city hospitals (which were multiservice, better equipped and had higher quality of ICU care) over that of the regional (rayon) hospitals contributed to better survival results for city residents.

Despite extensive preemptive measures and precautions taken in anticipation of the peak of COVID-19 pandemic, there was insufficient cumulative number of beds in provisional hospitals during the June–July 2020, with higher incidence of in-hospital and post-hospital deaths. One of the interesting findings is that patients treated at provisional hospitals (compared to infectious disease hospitals) had 1.9-fold and 3.8-fold higher risk of overall and post-hospital mortality, respectively. The level of financial and medical support provided by the government for the provisional and infectious diseases hospitals is unknown. We can only speculate that infectious diseases hospitals might have advantages in terms of newly equipped medical devices (lung ventilators, oxygen supplies), as well as better supply of medications which were suggested in the national protocol. On the other hand, the occupancy level of provisional hospitals was higher than infectious disease hospitals, especially during the peak period, which may have affected the quality of medical care and limited access to intensive care units in provisional hospitals [28, 29]. Also, 77% of all PCR-test positive cases, 2% of all PCR-test negative cases and 8% of all PCR-test unknown cases were treated at infectious diseases hospitals, and probably they stayed at the hospital until clinical improvement or death. Hence, the overall and post-hospital mortality rates at infectious diseases hospitals could be relatively low.

Initial recommendations of national guidelines on triaging patients to quarantine, provisional and infectious diseases hospitals based on their PCR-test results were very useful during the first few months of pandemic [16, 17]. However, an excessive number of PCR-test negative cases with similar COVID-19 symptoms overloaded the provisional hospitals. The possible reasons of PCR-test negativity could be false-negative results due to (i) inaccurate or low sensitivity test kits, (ii) incorrect nasopharyngeal swab technique, (iii) testing outside of the optimal time period (too early or too late) during low viral load and (iv) contamination of test samples [30–33]. Another potential reason might be a genetic diversity within species of SARS-CoV-2 [31, 34]. In the reported studies, the PCR-test false negative rate ranged from 2 to 50% [35, 36], however, in our data the PCR-test negative cases reached 94,5% among all PCR-tested cases. The update of national guidelines on 10th edition (June 29th, 2020), suggested triaging all COVID-19 suspected cases based on severity of clinical conditions (regardless of PCR-tests results) and to refer severely ill patients to provisional or ID-hospitals, and mild to moderate cases to outpatient services or multidisciplinary hospitals. By then it was too late, as the number of new referrals exceeded the bed capacity of most provisional hospitals in comparison to ID-hospitals during the peak of COVID-19 in June 20–July 20, 2020 period. This situation may have led to substantial medical care quality issues in provisional hospitals which was reflected on the high rate of in-hospital and overall mortality.

The worldwide infection fatality rate was reported around 0.68% (0.53–0.82%) across the population [37] and overall mortality rate from COVID-19 is around 2.3% and varies among countries [1]. In Kazakhstan, the overall mortality rate reported is 1.6% as of November 21st [38, 39]. Official statistic potentially underestimate mortality with uncaptured deaths from COVID-19 who died during post-discharge period, and people who could not get a medical attention and died at home or misdiagnosed. Our current data from the Turkestan region presented an in-hospital mortality rate of 1.3%, and overall (in-hospital and post-hospital) mortality rate of 3.0%. Several SARS-CoV-2 PCR-test negative patients discharged from the hospital without improvements, even with deterioration. Our post-hospital follow-up data showed that cumulative numbers of post-hospital deaths were higher than in-hospital deaths. Approximately 55 and 35 deaths were registered on post-discharge days 1 and 2, respectively. The explanation for that could be (i) the patients themselves or (ii) the relatives withdrew care or, (iii) patients were prematurely discharged from the provisional hospitals, despite the severity of their clinical conditions; (iv) deaths were registered in outpatient setting after hospital discharge or (v) daily electronic death reports were late due to extreme overload of workflow during the peak of COVID-19 pandemic.

There are several limitations worth mentioning. The distribution of patients based on PCR-test results taken from the available variables in the dataset, and all missing PCR-tests cases considered as “unknown”, although, these cases may have PCR-test results prior to their admission to the hospital. Moreover, we do not know the information about PCR-test kits (origin, producing companies, commercial names, sensitivity and validity, approval data), which might lead to false negative or positive results. The dataset includes all admissions with ICD-10 codes of respiratory diseases (J00-J00) and COVID-19 specific codes (B34, Z20, U07.1 and U07.2) for the period of March–July 2020, therefore, there might be several cases with different respiratory system diagnosis other than COVID-19. The survival and regression analyses are limited with available variables in the dataset, and the residual confounding is likely present in the study results as the current data was limited to few variables which could be adjusted for. The current data also limits lack of cause-specific mortality data, possible errors with disease coding, and lack of clinical variables such as severity of illness, laboratory parameters, oxygen saturation, radiologic evaluations (chest X-ray and computed tomography), and received treatment options. Another limitation occurs when reporting the case-fatality rate, which is based on reported incidence (not on true incidence) that could be heavily influenced by temporal changes due to available testing kits, how and when PCR-testing was conducted, how triage is conducted, etc.

Nonetheless, given the above limitations, this is the first study from Central Asia to demonstrate the peak of COVID-19 pandemic in one of the densely populated regions of Kazakhstan using digital healthcare data. We attempted to provide a full analysis of all hospital admissions including respiratory systems diseases and COVID-19 related ICD-10 codes, with analysis of in-hospital and post-hospital mortality during the peak of COVID-19 pandemic.

## Conclusion

This is the first study from the Central Asian and Eurasian region, evaluating the mortality of SARS-CoV-2 PCR-positive and PCR-negative respiratory system diseases admitted to the hospitals during the peak of COVID-19 pandemic. During the March–July 2020 period, the incidence and mortality rate from respiratory system diseases were 4-fold and 11-fold higher compared to the same period in 2019. The majority of hospitalized patients with pneumonia had PCR-test negative results, suggesting COVID-19-like pneumonia. The results showed extremely outnumbered admissions to provisional hospitals compared to infectious diseases hospitals with statistically significant higher mortality of patients treated in provisional hospitals. We describe a higher mortality rate for PCR-test positive cases compared to PCR-test negative cases, for males compared to females, for elder patients compared to younger ones and for patients living in rural areas compared to city residents. The findings of our study warrant further investigations and will help to make timely decisions for policy and decision makers.

## Supplementary Information

**Additional file 1 Supplement Table 1.** Association between in-hospital mortality and socio-demographic parameters using unadjusted (crude) and adjusted Cox proportional hazard regression analysis. **Supplement Table 2.** Association between post-hospital mortality and socio-demographic parameters using unadjusted (crude) and adjusted competing risk regression analysis. **Supplement Table 3.** Association between overall mortality (in-hospital and post-hospital) and socio-demographic parameters using unadjusted (crude) and adjusted Cox proportional hazard regression analysis in subgroup of patients admitted to provisional and infectious disease hospitals. **Supplement Table 4.** General characteristics of cohorts in 2019 and 2020. **Supplement Table 5.** Association between in-hospital mortality and socio-demographic parameters using unadjusted (crude) and adjusted Cox proportional hazard regression analysis (2019). **Supplement Figure 1.** Number of daily deaths after hospital discharge. **Supplement Figure 2.** Unadjusted in-hospital survival probability by SARS-CoV-2 PCR test results. **Supplement Figure 3**. Cumulative incidence of post-hospital mortality by SARS-CoV-2 PCR-test results (competing risk is in-hospital mortality).

## Data Availability

The data that support the findings of this study are available from Republican Center for Electronic Health of the Ministry of Health of the Republic of Kazakhstan, but restrictions apply to the availability of these data, which were used under the contract-agreement for the current study, and so are not publicly available. Data are however available from the authors upon reasonable request and with permission of Ministry of Health of the Republic of Kazakhstan.

## Abbreviations

CI: Confidence interval
COVID-19: Coronavirus Disease 2019
CT: Computed tomography
HR: Hazard ratio
ICD: International code of diseases
IQR: Interquartile ranges
PCR: Polymerase chain reaction
PMP: Per million population
PPE: Personal protective equipment
RPN-ID: Population registry number
SARS-CoV-2: Severe acute respiratory syndrome coronavirus-2
UNEHS: Unified National Electronic Health System
WHO: World Health Organization
