# Supplementary material for: SARS-CoV-2 PCR-positive and PCR-negative cases of pneumonia admitted to the hospital during the peak of COVID-19 pandemic: analysis of in-hospital and post-hospital mortality
Source: BMC Infect Dis. 2021 May 20;21:458. doi: 10.1186/s12879-021-06154-z (PMC8134816; doi:10.1186/s12879-021-06154-z)

**SUPPLEMENT MATERIALS**

**Title page**

**SARS-CoV-2 PCR-positive and PCR-negative cases of pneumonia admitted to the hospital during the peak of COVID-19 pandemic: analysis of in-hospital and post-hospital mortality**

**(Running title:** SARS-CoV-2 PCR-positive and PCR-negative pneumonias in Kazakhstan)

Abduzhappar Gaipov^1^, Arnur Gusmanov^1^, Anara Abbay^1^, Yesbolat Sakko^1^, Alpamys Issanov^1^, Kainar Kadyrzhanuly^1^, Zhanar Yermakhanova^2^, Lazzat Aliyeva^3^, Ardak Kashkynbayev^4^, Iklas Moldaliyev^5^, Byron Crape^1^, Antonio Sarria-Santamera^1^.

^1^Department of Medicine, Nazarbayev University School of Medicine, Nur-Sultan, Kazakhstan.

^2^Department of Emergency Medicine, Akhmet Yassawi University Medical Faculty, Turkestan, Kazakhstan.

^3^Department of expertise, Social Health Insurance Fund branch of the Turkestan region, Turkestan, Kazakhstan.

^4^Department of Mathematics, Nazarbayev University School of Sciences and Humanities, Nur-Sultan, Kazakhstan.

^5^Department of Preventive Medicine, Akhmet Yassawi University Medical Faculty, Turkestan, Kazakhstan.

**Corresponding author:** Abduzhappar Gaipov, MD, PhD, Assistant professor, Department of Medicine, Nazarbayev University School of Medicine. Address: Kerey and Zhanibek Khans Street 5/1, Room 345, Nur-Sultan city, Kazakhstan. Phone: +7 (7172) 70-62-97, e-mail: [abduzhappar.gaipov@nu.edu.kz](mailto:abduzhappar.gaipov@nu.edu.kz)

**Supplement Table 1.** Association between in-hospital mortality and socio-demographic parameters using unadjusted (crude) and adjusted Cox proportional hazard regression analysis.

| **In-hospital mortality** | | | | |
| --- | --- | --- | --- | --- |
| **Variable** | **Bivariate analysis** | | **Multivariable model** | |
|  | **crude HR (95% CI)** | **P value** | **adjusted HR (95%CI)*** | **P value** |
| **Sex**  Female  Male | Ref.  1.04 (0.79-1.34) | 0.77 | Ref.  1.24 (0.94-1.63) | 0.12 |
| **Age category**  <40  40-59  >=60 | Ref.  8.40 (4.19-16.82)  30.06 (15.29-59.12) | Ref.  <0.001  <0.001 | Ref.  8.5 (4.23-17.01)  30.1 (15.30-59.42) | Ref.  <0.001  <0.001 |
| **Residency**  City  Rural | Ref.  1.00 (0.76-1.31) | 0.99 | Ref.  0.87 (0.66-1.14) | 0.31 |
| **PCR-test result**  negative  positive  unknown | Ref.  1.90 (1.28-2.84)  2.11 (1.51-2.94) | Ref.  0.002  <0.001 | Ref.  1.72 (1.15-2.57)  1.73 (1.24-2.43) | Ref.  0.009  0.001 |
| **Hospital profile**  Infectious disease  Provisional | Ref.  1.26 (0.86-1.85) | 0.238 | - | - |

***** Adjusted for: Sex, age, residency, PCR-test results.

**Supplement Table 2.** Association between post-hospital mortality and socio-demographic parameters using unadjusted (crude) and adjusted competing risk regression analysis.

| **Post-discharge mortality (competing risk - in-hospital mortality)** | | | | |
| --- | --- | --- | --- | --- |
| **Variable** | **crude SHR (95% CI)** | **P value** | **adjusted SHR (95%CI)** | **p-value** |
| Sex  Female  Male | Ref.  0.96 (0.76-1.21) | 0.71 | Ref.  1.63 (0.98-1.56) | 0.07 |
| Age category  <40  40-59  >=60 | Ref.  7.26 (4.04-13.01)  34.06 (19.47-59.60) | Ref.  <0.001  <0.001 | Ref.  7.12 (3.94-12.85)  32.82 (18.64-57.81) | Ref.  <0.001  <0.001 |
| Residency  City  Rural | Ref.  2.18 (1.69-2.82) | <0.001 | Ref.  1.63 (1.26-2.11) | <0.001 |
| Test result  negative  positive  unknown | Ref.  0.66 (0.34-1.28)  0.88 (0.62-1.24) | Ref.  0.215  0.465 | Ref.  0.61 (0.32-1.18)  0.85 (0.61-1.20) | Ref.  0.145  0.364 |
| Profile  Infectious disease  Provisional | Ref.  3.88 (1.93-7.81) | <0.001 | - | - |

***** Adjusted for: Sex, age, residency, PCR-test results.

**Supplementary Table 3.** Association between overall mortality (in-hospital and post-hospital) and socio-demographic parameters using unadjusted (crude) and adjusted Cox proportional hazard regression analysis in subgroup of patients admitted to provisional and infectious disease hospitals.

| **Overall (combined in-hospital and post-hospital) mortality** | | | | |
| --- | --- | --- | --- | --- |
| **Variable** | **Bivariate analysis** | | **Multivariable model** | |
| **Variable** | **crude HR (95% CI)** | **P value** | **adjusted HR (95%CI)*** | **P value** |
| **Sex**  Female  Male | Ref.  1.21 (1.02-1.45) | 0.03 | Ref.  1.37 (1.14-1.63) | 0.001 |
| **Age category**  <40  40-59  >=60 | Ref.  7.56 (4.77-12.0)  29.22 (18.68-45.71) | Ref.  <0.001  <0.001 | Ref.  7.55 (4.76-11.98)  28.66 (18.29-44.90) | Ref.  <0.001  <0.001 |
| **Residency**  City  Rural | Ref.  1.39 (1.16-1.67) | <0.001 | Ref.  1.16 (0.97-1.40) | 0.106 |
| **PCR-test results**  negative  positive  unknown | Ref.  0.95 (0.69-1.32)  1.35 (1.07-1.70) | Ref.  0.781  0.012 | Ref.  2.32 (1.46-3.71)  1.42 (1.11-1.79) | Ref.  <0.001  0.004 |
| **Hospital profile**  Infectious disease  Provisional | Ref.  1.95 (1.41-2.70) | <0.001 | Ref.  2.62 (1.63-4.19) | <0.001 |

***** Adjusted for: Sex, age, residency, PCR-test results.

**Supplement Table 4.** General characteristics of cohorts in 2019 and 2020.

| **Variable** | **2019**  **(n= 4,600)** | **2020**  **(n=17,691)** | **p-value** |
| --- | --- | --- | --- |
| **Age, mean (**$\pm$**sd)** | 28.5 (23.1) | 43.1 (18.6) | <0.001¹ |
| **Age, N (%)**  <20  20-29  30-39  40-49  50-59  60-69  >69 | 1,670 (36.3)  1,090 (23.7)  595 (12.9)  329 (7.2)  304 (6.6)  298 (6.5)  314 (6.8) | 1,627 (9.2)  2,754 (15.6)  3,226 (18.2)  3,020 (17.1)  3,379 (19.1)  2,419 (13.7)  1,266 (7.2) | <0.001² |
| **Sex, N (%)**  Female  Male | 2,634 (57.3)  1,966 (42.7) | 8,617 (48.7)  9,074 (51.3) | <0.001² |
| **Residency, N (%)**  Rural  City | 3,186 (69.3)  1,414 (30.7) | 9,659 (54.6)  8,032 (45.4) | <0.001² |
| **Month of admission, N (%)**  March  April  May  June  July | 1,088 (23.6)  1,055 (22.9)  991 (21.5)  966 (21.0)  500 (10.8) | 264 (1.5)  2,820 (15.9)  3,644 (20.6)  5,757 (32.5)  5,206 (29.4) | <0.001² |
| **Number of days in hospital, median (IQR)** | 7 (5-9) | 3 (2-7) | <0.001¹ |
| **Clinical outcome at discharge**  Without change  Recovery  In-hospital death  Improvement  Deterioration | 69 (1.5)  2,614 (56.8)  24 (0.5)  1,891 (41.1)  2 (0.04) | 1,592 (9.0)  4,790 (27.1)  226 (1.3)  11,028 (62.3)  55 (0.3) | <0.001² |
| **Type of Healthcare organization**  City hospitals  Oblast hospitals  Rayon hospitals  Other med organizations  Non-medical organizations (temporary) | 595 (12.9)  114 (2.5)  3,821 (83.1)  70 (1.5)  0 (0.0) | 1,861 (10.5)  277 (1.6)  8,189 (46.3)  2,295 (13.0)  5,069 (28.7) | <0.001² |

¹ Two-sample t-test, ² Pearson’s Chi-squared test,

**Supplement Table 5.** Association between in-hospital mortality and socio-demographic parameters using unadjusted (crude) and adjusted Cox proportional hazard regression analysis (2019).

| **Variable** | **Bivariate analysis** | | **Multivariable model** | | |
| --- | --- | --- | --- | --- | --- |
|  | **crude HR (95% CI)** | **P value** | **adjusted HR (95%CI)*** | **P value** |  |
| **Sex**  Female  Male | Ref.  1.93 (0.82-4.51) | 0.13 | Ref.  1.86 (0.79-4.36) | 0.15 |  |
| **Age category**  <40  40-59  >=60 | Ref.  20.59 (4.37-97.01)  30.92 (6.9-138.4) | Ref.  <0.001  <0.001 | Ref.  21.45 (4.55-101.09)  29.08 (6.48-130.45) | Ref.  <0.001  <0.001 |  |
| **Residency**  City  Rural | Ref.  2.79 (0.83-9.44) | Ref.  0.1 | Ref.  2.75 (0.81-9.35) | Ref.  0.1 |  |

***** Adjusted for: Sex, age, residency.

**Supplement Figure 1.** Number of daily deaths after hospital discharge.


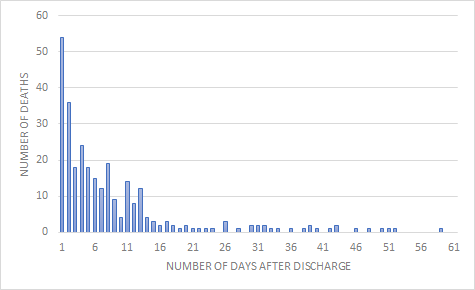


**Supplement Figure 2.** Unadjusted in-hospital survival probability by SARS-CoV-2 PCR test results.


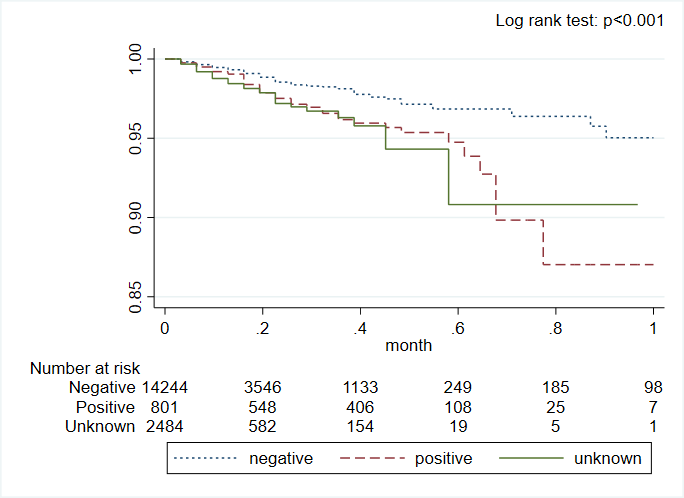


**Supplement Figure 3**. Cumulative incidence of post-hospital mortality by SARS-CoV-2 PCR-test results (competing risk is in-hospital mortality).


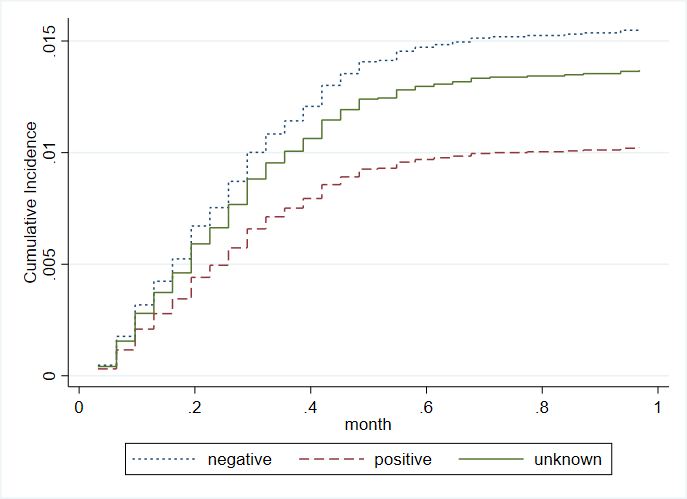

Supplement: Supplementary file 1 — Additional file 1 Supplement Table 1. Association between in-hospital mortality and socio-demographic parameters using unadjusted (crude) and adjusted Cox proportional hazard regression analysis. Supplement Table 2. Association between post-hospital mortality and socio-demographic parameters using unadjusted (crude) and adjusted competing risk regression analysis. Supplement Table 3. Association between overall mortality (in-hospital and post-hospital) and socio-demographic parameters using unadjusted (crude) and adjusted Cox proportional hazard regression analysis in subgroup of patients admitted to provisional and infectious disease hospitals. Supplement Table 4. General characteristics of cohorts in 2019 and 2020. Supplement Table 5. Association between in-hospital mortality and socio-demographic parameters using unadjusted (crude) and adjusted Cox proportional hazard regression analysis (2019). Supplement Figure 1. Number of daily deaths after hospital discharge. Supplement Figure 2. Unadjusted in-hospital survival probability by SARS-CoV-2 PCR test results. Supplement Figure 3. Cumulative incidence of post-hospital mortality by SARS-CoV-2 PCR-test results (competing risk is in-hospital mortality). [file 12879_2021_6154_MOESM1_ESM.docx]
